# Trends in delirium coding rates in older hospital inpatients in England and Scotland: full population data comprising 7.7M patients per year show substantial increases between 2012 and 2020

**DOI:** 10.56392/001c.84051

**Published:** 2023-07-29

**Authors:** Temi Ibitoye, Thomas A. Jackson, Daniel Davis, Alasdair M.J. MacLullich

**Affiliations:** 1 Edinburgh Delirium Research Group, Ageing and Health, Usher Institute, The University of Edinburgh; 2 Institute of Inflammation and Ageing, University of Birmingham; 3 MRC Unit for Lifelong Health and Ageing, University College London; 4 Edinburgh Delirium Research Group, Ageing and Health, Usher Institute, University of Edinburgh

**Keywords:** Coding, Delirium, ICD-10, Geriatric Medicine, Hospital Administrative Database, National Data

## Abstract

**Background:**

Little information is available on change in delirium coding rates over time in major healthcare systems. We examined trends in delirium discharge coding rates in older patients in hospital admissions to the National Health Service (NHS) in England and Scotland between 2012 and 2020.

**Methods:**

Hospital administrative coding data were sourced from NHS Digital England and Public Health Scotland. We examined rates of delirium (F05 from ICD-10) in patients aged ≥70 years in 5 year and ≥90 age bands.

**Results:**

There were approximately 7,000,000 discharges/year in England and 700,000/year in Scotland. Substantially increased delirium coding was observed for all age bands between 2012/2013 and 2019/2020 (p<0.001, Mann Kendall’s tau). In the ≥90 age band, there was a 4-fold increase between 2012 and 2020.

**Conclusion:**

Delirium coding rates have shown large increases in the NHS in England and Scotland, likely reflecting several factors including policy initiatives, detection tool implementation and education.

## Introduction

Delirium is an acute and common condition characterised by disturbances in attention, awareness and cognition, that affects roughly 23% of older inpatients.^1^ Patients with delirium are at a higher risk of adverse health outcomes, including increased risk of death, cognitive decline, and prolonged hospital length of stay.^2,3^ Despite the known consequences of delirium, current studies suggest that it is grossly under-reported in medical records, including discharge summaries and hospital administrative systems.^4^

Discharge summaries are a vital communication pathway between primary, secondary and tertiary healthcare providers. Information recorded in the discharge summaries is used by hospital administrative coders, in conjunction with inpatient medical records, to translate medical diagnoses into a standard coded format, such as the International Classification of Diseases - Tenth Revision (ICD-10).^5^ The 5^th^ edition of ICD-10 was implemented across NHS England and NHS Scotland around 1^st^ April 2016.^6,7^ Coding generates data used for clinical governance and informs policy-makers. It is used for statistics, hospital reimbursement, resource allocation and research services.^8^ Therefore, inaccurate reporting, or the lack of reporting, of delirium in discharge summaries and hospital administrative systems has significant adverse consequences for patient continuity of care (e.g. appropriate follow-up in the community for memory assessments) and healthcare service planning.

Despite being highly prevalent in older adult inpatients, delirium detection remains an ongoing clinical problem. The key challenge of providing robust delirium detection in routine care has been addressed in multiple guidelines in healthcare systems internationally. In the United Kingdom (UK), the 2010 National Institute for Health and Care Excellence (NICE) and Scottish Intercollegiate Guidelines Network (SIGN) delirium guidelines both advocate that delirium detection tools, such as the 4 A’s Test (4AT; www.the4AT.com), are integrated into routine care.^9–11^ However, little information is available regarding the impact of these guidelines on recording of delirium diagnoses across the whole healthcare system.

Here we examined trends in discharge coding rates in older patients from all hospital admissions to the National Health Service (which provides >90% of care to the whole population) in England and Scotland, between 2012 and 2020.

## Methods

Statistics for annual hospital admissions for patient care activities were sourced from the NHS Digital England website^12^ and via a request to Public Health Scotland. We examined proportions of delirium coded as F05 in ICD-10 (delirium, not induced by alcohol and other psychoactive substances), designated as ‘any diagnosis’ (present as a primary or non-primary diagnosis).

Data were analysed using RStudio version 4.1.2.^13^ We calculated the prevalence by dividing the total number of patients with F05 diagnostic discharge code by the total number of hospital admissions for each age band for each financial year (Table 1). We tested for time trends in five-year and ≥90 age bands using Mann-Kendall’s tau test.

## Results

**Figure 1:**
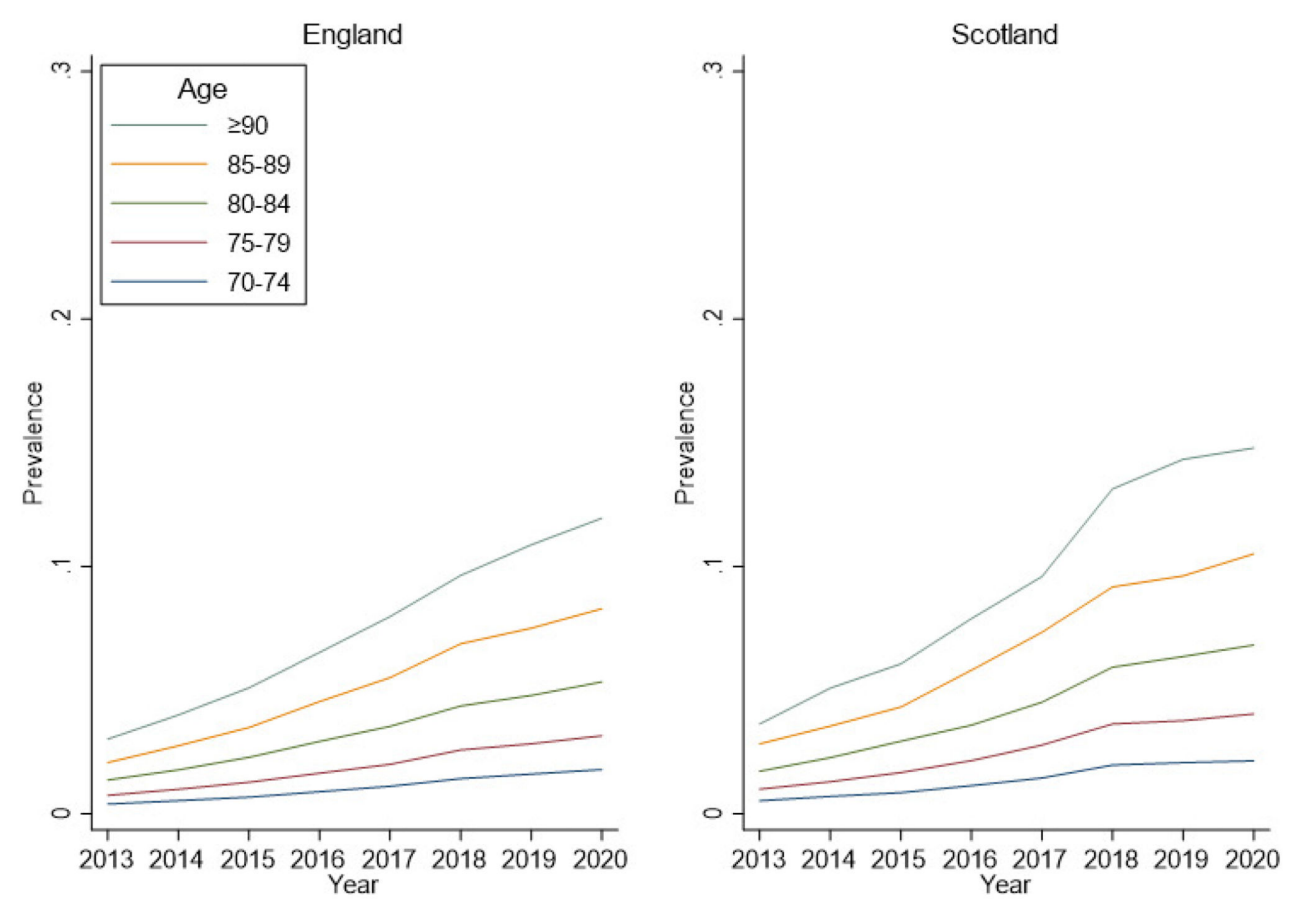
Hospital delirium prevalence in England and Scotland between 2012/13 and 2019/20 Prevalence expressed as a fraction of 1.

The population data comprised approximately 7,000,000 discharges annually in England and 700,000 annually in Scotland (Table 1). Delirium coding in older inpatients increased between 2012/2013 and 2019/2020 (p<0.001) (Figure 1).

The most substantial increases were seen in the older age groups, with 4-5 fold increases in coding in the ≥90 age group and 3-4 fold increases in the 85-89 age group. In 2012/2013, for patients aged ≥90 18,137 (3%) patients in England and 1,671 (3.6%) patients in Scotland were discharged with an *any delirium* diagnostic code (Table 1). This contrasts with 97,678 (12%) patients in England and 9,227 (14.8%) patients in Scotland with delirium diagnostic codes for the year 2019/2020 at the same age.

## Discussion

We analysed changes in delirium discharge coding rates over time, finding significantly increased trends at all older ages between 2012 and 2020. The increases in coding rates were proportionally more pronounced in the older age groups (80-84, 85-89 and ≥90, Figure 1). The overall increasing trends could be due to better recognition of delirium and documentation in discharge summaries since robust delirium detection methods (such as the 4AT) have been widely implemented across the UK.^9^ National guidelines have led to an upsurge in educational and audit initiatives. Together, these have encouraged more consistent use of the term *delirium* in favour of outdated nomenclature such as *acute confusion*.^10,14^ When delirium is properly documented in discharge summaries, the accuracy of ICD-10 delirium coding in hospital administrative systems is increased.^15^ These data report up until the end of March 2020, which coincides with the start of the Covid-19 pandemic in the UK.

Though the trends observed are encouraging, the rates of delirium coding remain considerably below the true rates measured in previous studies, which show that around a quarter of older inpatients have delirium on admission or soon after.^1^ This suggests that, while apparent progress has been made in the UK to tackle this ongoing challenge, delirium remains under-coded.

These findings emphasise the importance of continuous and effective delirium training for healthcare workers. When such measures are put in place this can improve delirium detection, documentation in discharge summaries and, ultimately, discharge coding. For example, in a prospective cohort study looking at delirium coding trends, Pendlebury et al. (2020) reported that implementing a system-wide multicomponent intervention consisting of delirium screening, training, educational seminars, and audits increased hospital ICD-10 delirium coding sensitivity by roughly six-fold over an eight-year period.^16^

This is the first publication to report delirium coding trends using national full population data. The study benefits from a very large sample size across England and Scotland, and uses real-world data from whole unselected populations. Examining delirium coding rates including trends across major healthcare systems is a useful indicator of the overall performance of such systems in detecting delirium, and could be applied in multiple other national and organisational settings.

## Figures and Tables

**Table 1:** Delirium prevalence rates in England and Scotland between 2012/13 and 2019/20

**NHS England *n/N (%)***					
Age Bands (years)	70 - 74	75 - 79	80 - 84	85 - 89	90+
Financial Year					
**2012/13**	5,300/1,342,406 (0.4)	10,488/1,398,795 (0.7)	17,589/1,286,768 (1.4)	19,694/950,168 (2.1)	18,137/600,678 (3.0)
**2013/14**	7,427/1,399,136 (0.5)	14,490/1,460,814 (1.0)	23,626/1,329,111 (1.8)	26,835/975,464 (2.8)	24,718/618,912 (4.0)
**2014/15**	10,013/1,481,483 (0.7)	19,690/1,542,220 (1.3)	31,950/1,399,036 (2.3)	36,229/1,038,266 (3.5)	34,280/673,088 (5.1)
**2015/16**	13,864/1,550,780 (0.9)	25,879/1,575,181 (1.6)	41,536/1,419,823 (2.9)	48,385/1,066,297 (4.5)	44,871/687,445 (6.5)
**2016/17**	18,474/1,657,098 (1.1)	32,050/1,602,659 (2.0)	51,936/1,468,328 (3.5)	61,453/1,116,309 (5.5)	57,853/725,408 (8.0)
**2017/18**	25,323/1,778,841 (1.4)	42,241/1,635,310 (2.6)	66,076/1,515,412 (4.4)	80,008/1,164,106 (6.9)	73,376/761,012 (9.6)
**2018/19**	30,576/1,902,352 (1.6)	48,812/1,722,878 (2.8)	76,646/1,600,911 (4.8)	89,992/1,199,464 (7.5)	84,925/780,945 (10.9)
**2019/20**	34,757/1,939,898 (1.8)	56,713/1,798,153 (3.2)	87,933/1,648,069 (5.3)	102,073/1,230,837 (8.3)	97,678/817,165 (12.0)
**NHS Scotland n/N (%)**					
**2012/13**	740/139,948 (0.5)	1,405/141,038 (1.0)	2,117/123,119 (1.7)	2,326/82,202 (2.8)	1,671/45,980 (3.6)
**2013/14**	1,023/145,403 (0.7)	1,959/150,715 (1.3)	2,916/128,344 (2.3)	3,093/87,185 (3.5)	2,490/49,037 (5.1)
**2014/15**	1,299/151,636 (0.9)	2,605/155,937 (1.7)	4,000/136,329 (2.9)	3,973/92,047 (4.3)	3,253/53,674 (6.1)
**2015/16**	1,776/156,038 (1.1)	3,430/159,362 (2.2)	5,079/141,623 (3.6)	5,632/96,980 (5.8)	4,335/54,933 (7.9)
**2016/17**	2,357/162,665 (1.4)	4,468/160,804 (2.8)	6,555/145,279 (4.5)	7,438/101,336 (7.3)	5,618/58,577 (9.6)
**2017/18**	3,350/169,993 (2.0)	5,840/160,762 (3.6)	8,495/143,316 (5.9)	9,428/102,812 (9.2)	7,849/59,746 (13.1)
**2018/19**	3,646/176,298 (2.1)	6,078/161,316 (3.8)	9,184/144,407 (6.4)	9,751/101,383 (9.6)	8,311/57,997 (14.3)
**2019/20**	3,906/182,457 (2.1)	6,739/166,796 (4.0)	10,231/149,912 (6.8)	11,297/107,476 (10.5)	9,227/62,404 (14.8)

All age trends were significant (p<0.001) using Kendall-Tau’s test. n is the number of patients with any “delirium” diagnostic discharge code. N is the population. % is the prevalence of delirium for each age band for that financial year.
